# Performance evaluation of a ring-worn pulse oximeter for the identification and monitoring of obstructive sleep apnea

**DOI:** 10.3389/frsle.2025.1549272

**Published:** 2025-04-15

**Authors:** Laura K. Gell, Ketan Mehta, Neda Esmaeili, Luigi Taranto-Montemurro, Scott A. Sands, Stephen D. Pittman, Ali Azarbarzin

**Affiliations:** ^1^Apnimed Inc., Cambridge, MA, United States; ^2^Division of Sleep and Circadian Disorders, Brigham and Women's Hospital and Harvard Medical School, Boston, MA, United States

**Keywords:** oximetry, validation, obstructive sleep apnea, wearable, monitoring

## Abstract

**Introduction:**

Obstructive sleep apnea (OSA) is a highly prevalent chronic disorder that is challenging to monitor clinically. While single-night laboratory-based polysomnography (PSG) is the current gold standard for OSA assessment, its utility is limited by cost and inaccessibility. Overnight pulse oximetry is a feasible approach for simplified at-home monitoring of OSA. In this study, we evaluate the performance of a modified finger-worn pulse oximetry device (“Ring”) for OSA assessment.

**Methods:**

In all, 25 patients with OSA [age: 55.5 ± 7.7 years (mean ± *SD*), body mass index (BMI): 31.8 ± 5.1 kg/m^2^, 14M:11F, and Fitzpatrick scale score I–II: 15, III–IV: 6, and V–VI: 4] completed up to four in-laboratory PSG studies with simultaneous Ring oximetry measurements (90 studies in total). Correlation and agreement analyses compared Ring-derived measures of the oxygen desaturation index (ODI4_RING_, desaturations ≥4%) against PSG measures (ODI4_PSG_ and AHI4_PSG_). Likewise, Ring-derived hypoxic burden (HB_RING_) was compared against its PSG counterpart (HB_PSG_). Receiver operator characteristic (ROC) curve analysis quantified the ability of ODI4_RING_ to identify moderate-to-severe OSA (AHI4_PSG_ > 15 events/h).

**Results:**

Median [interquartile range (IQR)] of AHI4_PSG_ was 18.0 [9.6, 31.7] events/h. ODI4_RING_ was positively correlated with ODI4_PSG_ (Pearson *r* = 0.87, root mean square error [RMSE] = 6.6 events/h, intraclass correlation [ICC] = 0.85) and AHI4_PSG_ (*r* = 0.85, RMSE = 7.1 events/h, ICC = 0.84). The bias (mean difference) and limits of agreement (1.96 *SD*) between ODI4_PSG_ and ODI4_RING_ were 2.9 and 14.2 events/h, while for AHI4_PSG_ and ODI4_RING_, the bias and limits of agreement were 1.4 and 16.3 events/h, respectively. HB_RING_ was positively correlated with HB_PSG_ (*r* = 0.75, RMSE = 24.6% min/h, ICC = 0.73), with a mean difference of 3.7% min/h and limits of agreement of 60.6% min/h. The receiver operator characteristic curve analysis of ODI4_RING_ to identify moderate-to-severe OSA produced an area under the curve of 0.92 (ODI4_PSG_ > 15 events/h, “excellent”) and 0.84 (AHI4_PSG_ > 15 events/h, “excellent”).

**Conclusion:**

Our results show that a low-cost, convenient, and simple-to-use finger-worn pulse oximeter is a reliable tool for continuous monitoring of OSA severity and therapy responses. It also offers excellent discriminative value for screening moderate-to-severe OSA in this population.

## 1 Introduction

Obstructive sleep apnea (OSA) is the most common sleep-related breathing disease, estimated to affect approximately one in four adults (Benjafield et al., 2019; Heinzer et al., 2015), in which decreased upper airway muscle tone during sleep leads to a repeating cycle of partial airway collapse (hypopnea) and total obstruction (apnea) accompanied by oxygen desaturation and sleep fragmentation (White, 2005). Untreated OSA is associated with increased daytime sleepiness and fatigue, and increased risk of cardiovascular and cerebrovascular disease, metabolic dysfunction, and early mortality (Antic et al., 2011; Azarbarzin et al., 2018; Javaheri et al., 2017; Marin et al., 2005; Bonsignore et al., 2013; Marshall et al., 2008). Although awareness about the high prevalence of OSA in the community is increasing, it frequently remains undiagnosed, even in patients with moderate-to-severe disease. Currently, an in-laboratory polysomnography (PSG) sleep study is considered the gold standard tool for diagnosing OSA; however, it is costly and resource-intensive, and thus access is limited. Home sleep testing (HST) has become more common as an alternative to in-lab PSG, using devices that record at a minimum airflow, pulse rate, and pulse oximetry channels. Devices and sensors must still be applied by an experienced professional or under their supervision, and studies are scored manually by a registered technologist. Thus, despite alleviating some of the resource and patient burden, delays to diagnosis remain high, and it is estimated that the majority of those with OSA are undiagnosed and thus untreated (Heinzer et al., 2015). Therefore, the need for a simple and easy-to-use screening tool for OSA that could help identify those with undiagnosed or suspected OSA is significant.

Moreover, despite the chronic nature of OSA, clinicians rely on PSG or HST measurement of OSA severity derived from a single night to make treatment decisions. However, the known within-subject night-to-night variability in respiratory events makes this problematic. It has been estimated that single-night PSG studies lead to misdiagnosis between 20 and 60% of the time (Roeder et al., 2020; Punjabi et al., 2020; Skiba et al., 2015; Tschopp et al., 2021; Lechat et al., 2022), whereas multi-night monitoring reduces the likelihood of misdiagnosis (Lechat et al., 2022). More importantly, PSG and HST with multiple channels (type 3) are not well suited for serial assessments during therapy titration. Simple, reliable tools are needed to objectively quantify changes in OSA severity over time and evaluate responses to therapy. As more treatment options emerge for patients, including the recent approval of pharmacotherapy for OSA (Malhotra et al., 2024), the value of such insights will increase. An incomplete treatment response could indicate the need to modify therapy or dose/level or consider a combination of therapies or interventions; however, clinical tools to facilitate ongoing monitoring are currently lacking.

Single-channel pulse oximetry to determine the oxygen desaturation index (ODI) has been shown to be a useful screening tool for OSA severity (Chiner et al., 1999; Dumitrache-Rujinski et al., 2013) that could be used by patients at home and over multiple nights for continued monitoring of OSA; however, accuracy varies across devices (Rashid et al., 2021). In this study, we evaluate using a new modified, wearable finger-worn Ring pulse oximeter device combined with custom algorithms for use in the ongoing monitoring of OSA severity during a randomized crossover trial across different therapeutic conditions. In the primary analysis, the bias and limits of agreement between Ring and gold-standard PSG measurements of OSA severity (ODI4 and AHI4) were assessed. The secondary analysis assessed the intraclass and Pearson correlations between metrics and the ability of Ring ODI4 to identify moderate-to-severe ODI4. An exploratory analysis assessed the agreement between novel hypoxic burden measurements derived from the Ring oximeter alone and standard PSG measures.

## 2 Materials and methods

### 2.1 Study participants and design

This Ring oximetry ancillary study was conducted as part of a larger study (NCT05793684), approved by the WIRB-Copernicus Group Institutional Review Board (Aishah et al., 2024). Twenty-five participants with mild-to-severe OSA met eligibility criteria and were randomly assigned to the parent study across three sites: Brigham and Women's Hospital, Boston, Massachusetts (*N* = 1); Clayton Sleep Institute, St. Louis, Missouri (*N* = 14), and Santa Monica Clinical Trials, Los Angeles, California (*N* = 10). The exclusion criteria included clinically significant cardiac disease, neurological disorders, non-OSA sleep disorders, or uncontrolled hypertension. All participants provided informed written consent prior to study participation. During the study, participants completed up to four in-laboratory PSG studies under different treatment conditions (baseline, placebo, and treated with 500 mg Viloxazine, 500/75 mg Viloxazine-Trazadone). In total, 90 in-laboratory PSG studies were performed, and simultaneous Ring oximetry measurements were recorded successfully on all PSG nights.

### 2.2 Sleep study measurements

In-laboratory PSGs included measurements of electroencephalogram (EEG), electrooculogram (EOG), nasal pressure, thermistor, body position, and pulse oximetry using a finger probe. Oxygen saturation from PSG oximetry was sampled at a minimum rate of 1 Hz (10 Hz preferred), the signal averaging window was required to be between 1 and 3 s. In-lab PSGs were scored by a centralized PSG center, following the American Academy of Sleep Medicine (ASSM; Berry et al., 2017), with access to all PSG channels but blinded to the Ring oximetry data. The apnea–hypopnea index (AHI) was based on the AASM rule 1B for identification of hypopneas (which specifies a ≥ 30% reduction in airflow for ≥10 s and oxygen desaturation of ≥4%, AHI4_PSG_), as has been used recently to determine eligibility in several prominent OSA clinical trials (Malhotra et al., 2024; Schweitzer et al., 2023). We also measured the ODI based on ≥4% desaturation (ODI4_PSG_) and hypoxic burden (HB, area under the desaturation curve), based on manually scored respiratory events (Azarbarzin et al., 2018). Participants also wore a finger-based Ring pulse oximeter (Product Name: Pulse Oximeter, Model: S9; Shenzhen Viatom Technology Co. Ltd., Shenzhen, China) with modified firmware to meet our requirements. Oxygen saturation from the Ring was recorded at 1 Hz; oxygen desaturation from the Ring oximeter was determined as the number of desaturations ≥4% from baseline per hour over total recording time using custom algorithms (ODI4_RING_). HB from the Ring oximeter was also calculated (HB_RING_), as the area under the desaturation curve based on automatically detected oxygen desaturations ≥2% as described and validated by Esmaeili et al. (2023), divided by the total recording time.

### 2.3 Statistical analysis

The primary outcome was the bias and limits of agreement between ODI4_RING_ and ODI4_PSG_. Repeated linked measurements under different conditions within subjects may lead to an underestimation of the limits of agreement if standard Bland–Altman analyses (in which it is assumed measurements are independent) are used. To account for this, we instead implemented a mixed-model approach, as described by Carstensen et al. (2008), including the subject-by-oximetry method and subject-by-treatment interactions to ensure the variance is correctly attributed between terms. This mixed-model approach provides a modestly more conservative limit of agreement estimate than simple difference variance reporting because we are modeling how much the difference variance appears to be reduced by the repeated non-independent estimates within subjects. The mean difference was then determined from the oximetry method coefficient and limits of agreement by the formula 1.96 × √(2 × τ^2^ + $\sigma_{\text{PSG}}^{2}$ + $\sigma_{\text{RING}}^{2}$), where τ^2^ is the oximetry method by subject interaction variance and $\sigma_{\text{RING}}^{2}$ and $\sigma_{\text{PSG}}^{2}$ are the respective within-oximetry method residual variances. Agreement and limits were displayed on Bland–Altman plots for interpretation. The analysis was repeated to compare ODI4_RING_ and AHI4_PSG._ For secondary outcomes, the intraclass correlation coefficients (ICCs) were also determined between measurements (0.75–0.9: good reliability, >0.9: excellent reliability). Pearson correlation analysis was used to assess the strength of the linear relationship between the Ring and gold-standard PSG measurements and the spread of observed data around this model using the root mean square error (RMSE). A correlation coefficient, *r*, >0.7 is considered a strong correlation, with a minimum RMSE requirement of fewer than 10 events/h (i.e., the difference between “mild” and “moderate” OSA classification). Receiver operator characteristic (ROC) curve analyses and area under the curve (AUC) analyses were used to evaluate the identification of moderate-to-severe OSA using the Ring with a cutoff of AHI4_PSG_ > 15 and ODI4_PSG_> 15. An AUC >0.8 (“excellent”) was considered sufficient sensitivity and specificity for screening purposes. Optimal thresholds for identifying moderate-to-severe OSA were determined from the optimal operating point of the ROC curve, minimizing the misclassification cost where, given the intended use as a prescreening tool, false negatives were given twice the cost weighting of false positives. Pointwise confidence intervals for AUC, sensitivity, and specificity were calculated using bootstrapping with 1,000 iterations. In an exploratory analysis, we repeated the previously described analyses to assess the agreement between Ring- and PSG-derived hypoxic burden measurements; in ROC analyses, the ability of the Ring oximeter to screen for high HB (HB_PSG_ > 60) was evaluated. In the *post-hoc* subgroup analysis, descriptive statistics are reported to describe the bias in different subgroups using the Fitzpatrick scale for skin tone. Statistical analyses were performed using MATLAB (Natick, MA) and R version 4.3.2.

## 3 Results

**Figure 1:**
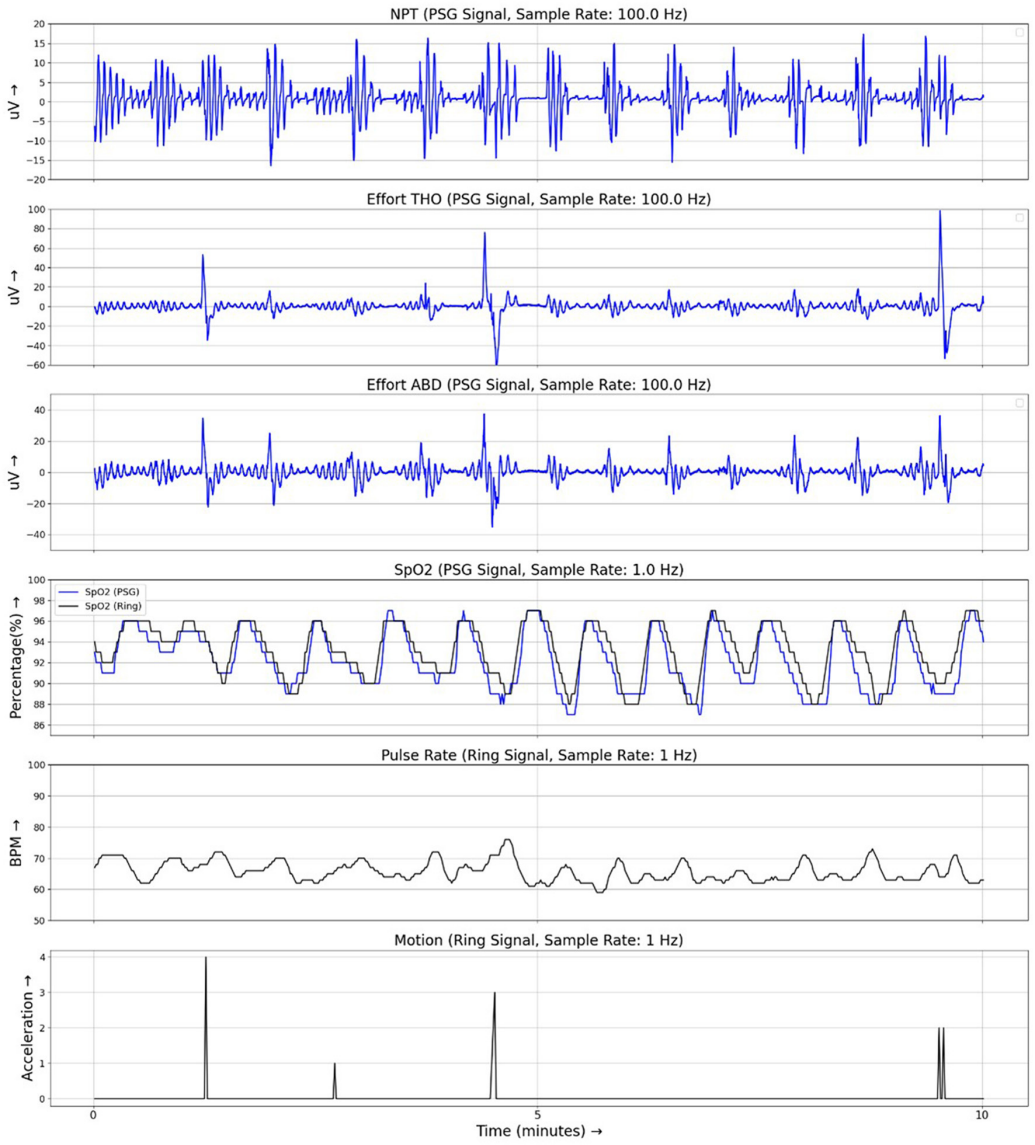
Example traces from simultaneous recordings of PSG and Ring data during a 10-min period of respiratory events. PSG signals are shown in blue and Ring signals are shown in black. NPT, nasal pressure; Effort THO, respiratory effort signal from the thoracic belt; Effort ABD, respiratory effort signal from the abdominal belt; SpO_2_, oximetry signal.

Patient characteristics are given in Table 1. The median [interquartile range (IQR)] AHI4_PSG_ was 18.0 [9.6, 31.7] events/h and ODI4_PSG_ 19.5 [12.7, 32.5] events/h. Of the 90 sleep studies, 55 exhibited moderate-to-severe OSA (AHI4 > 15) per PSG scoring, 25 exhibited mild OSA (5 ≤ AHI4 < 15), and 10 exhibited no OSA (AHI4 < 5). Ring analysis showed a median ODI4_RING_ of 16.8 (10.7, 26.9) events/h. The maximum data rejection rate for the Ring SpO_2_ data (null values as a percentage of total data points) across all recordings was 0.13%. Example signal traces from PSG and Ring recordings are shown in Figure 1.

**Table 1:** Patient characteristics.

**Demographics and obesity**	
Age (years)	56 [50–62]
Sex, *N* (M:F)	14:11
BMI (Kg/m^2^)	32 [28–38]
Race, *N* (Black:White:Asian:Other)	3:18:1:3
Ethnicity, *N* (Hispanic:Non-Hispanic)	3:22
Fitzpatrick scale (I:II:III:IV:V:VI)	3:12:5:1:3:1
**Polysomnography**	
Total sleep time (min)	387.3 [353.0, 424.0]
AHI4 (events/h)	18.0 [9.6, 31.7]
ODI4 (events/h)	19.5 [12.7, 32.5]
Hypoxic burden (%.min/h)	62.9 [41.2, 91.2]
Arousal index (events/h)	28.8 [19.6, 37.6]
Stage 1 (%TST)	12.2 [7.4, 17.0]
Stage 2 (%TST)	65.9 [60.8, 72.4]
Stage 3 (%TST)	8.7 [2.9, 14.0]
REM (%TST)	9.4 [4.6, 15.5]
T90 (%TST)	3.4 [0.8, 9.3]
Epworth sleepiness scale	9.5 [6.0, 12.0]
**Ring metrics**	
Total recording time (min)	491 [487.3, 496.7]
ODI4_RING_ (events/h)	16.8 [10.7, 26.9]
HB_RING_ (%.min/h)	59.7 [38.6, 83]

The Fitzpatrick scale is a descriptive classification of skin tone, with I being the lightest and VI being the darkest tone. Data are presented as median [interquartile range].

AHI4, apnea-hypopnea index with 4% oxygen desaturation; HB, hypoxic burden; ODI4, oxygen desaturation index with 4% oxygen desaturation; T90, sleep time under 90% oxygen desaturation; TST, total sleep time; REM, rapid eye movement; BMI, Body Mass Index.

**Figure 2:**
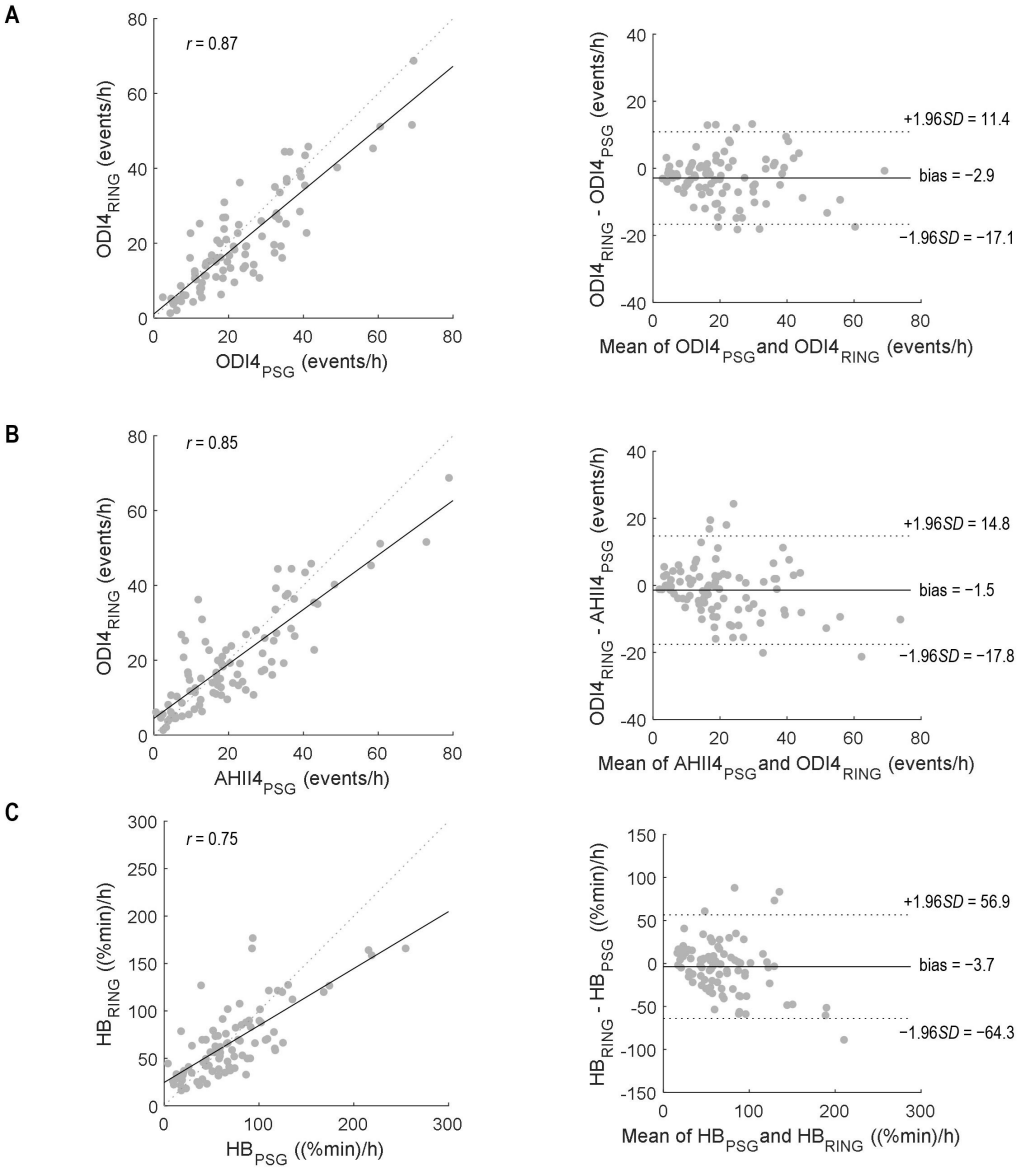
Correlation **(left)** and Bland–Altmann **(right)** plots to compare **(A)** ODI4_RING_ and ODI4_PSG_, **(B)** ODI4_RING_ and AHI4_PSG_, and **(C)** HB_RING_ and HB_PSG_. *r*, Pearson correlation coefficient. Bias and limits of agreement were calculated using mixed model analysis to account for within-subject repeated measurements. PSG, polysomnography.

**Figure 3:**
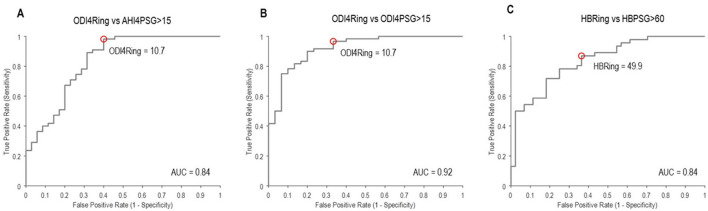
Receiver operator curves to identify moderate-to-severe OSA using **(A)** ODI4_RING_, as defined by AHI4_PSG_ ≥ 15 events/h; **(B)** ODI4_RING_, as defined by ODI4_PSG_ ≥ 15 events/h; and **(C)** HB_RING_, as defined by HB_PSG_ ≥ 60%min/h. AUC, area under the curve, >0.8 = “excellent”. The red circles denote the optimal cutoff point in ODI4_RING_ or HB_RING_ at the associated sensitivity and false positive (1 – specificity) values for screening, corresponding to **(A)** ODI4_RING_ = 10.7 events/h, **(B)** ODI4_RING_ = 10.7 events/h, and **(C)** HB_RING_ = 49.9%min/h.

From mixed-model analyses, the bias (mean difference) and limits of agreement between ODI4_PSG_ and ODI4_RING_ were 2.9 ± 14.2 events/h (Figure 2A), and between AHI4_PSG_ and ODI4_RING_, they were 1.5 ± 16.3 events/h (Figure 2B), respectively. We note that limits of agreement were only slightly underestimated when ignoring the effects of repeated measurements using standard Bland–Altman analyses (±13.8 and ±16.2 events/h, respectively), suggesting that correlations between within-subject repeated measures in different treatment conditions are low. ODI4_RING_ was positively correlated with ODI4_PSG_ (Pearson *r* = 0.87, 95% CI [0.81, 0.91], RMSE = 6.6 events/h; Figure 2A), and AHI4_PSG_ (*r* = 0.85 [0.78–0.90], RMSE = 7.1 events/h; Figure 2B). The ICC for ODI4_RING_ vs. ODI4_PSG_ and vs. AHI4_PSG_ was 0.85 (95% CI [0.78, 0.90]) and 0.84 [0.76–0.89], respectively, and for the ROC curve analysis of ODI4_RING_ to predict moderate-to-severe OSA produced an area-under-curve (AUC) of 0.84 (95% CI [0.73, 0.92]; AHI4_PSG_ >15 events/h, “excellent”, Figure 3A) and 0.92 [0.84–0.97] (ODI4_PSG_ >15 events/h, “excellent”; Figure 3B). The optimal cutoff for the screening of moderate-to-severe OSA by AHI4_PSG_ > 15 events/h (i.e., the gold standard) was determined to be ODI4_RING_ = 10.7 events/h, which was associated with a sensitivity of 0.98 [0.91–1.00] and a specificity of 0.60 [0.42–0.74] (Figure 3A). The optimal cutoff for the screening of moderate-to-severe OSA by ODI4_PSG_ > 15 events/h was determined to be ODI4_RING_ = 10.7 events/h, which was associated with a sensitivity of 0.97 [0.88–1.00] and a specificity of 0.67 [0.47–0.81] (Figure 3B). In the exploratory analysis, HB from the Ring oximetry, HB_RING_, was positively correlated with HB_PSG_ (*r* = 0.75 [0.65–0.83], RMSE = 24.6%min/h; Figure 2C), with a mean difference of 3.7%min/h and limits of agreement of ±60.6%min/h (Figure 2C). The ICC for HB_RING_ vs. HB_PSG_ was 0.73 [0.62–0.81], ROC analysis using HB_RING_ to predict moderate-to-severe HB_PSG_ > 60%min/h, AUC = 0.84 [0.74–0.91], and optimal threshold was determined to be 49.9%min/h, associated with a sensitivity of 0.87 [0.75–0.94] and specificity of 0.64 [0.47–0.76] (Figure 3C).

## 4 Discussion

This study has shown that the Ring oximeter device combined with custom algorithms can reliably detect OSA events and monitor OSA severity. The mean differences between ODI4_RING_ and PSG measurements were small, the ring slightly underestimates the severity of OSA on average (−2.9 events/h vs. AHI4_PSG_, −1.4 events/h vs. ODI4_PSG_). A visual analysis of the Bland–Altman plot suggests that bias tends to be greater at high values of ODI4. The limits of agreement of ODI4_RING_ with AHI4_PSG_ and with ODI4_PSG_ were ±14.2 events/h and ±16.3 events/h, respectively: 95% of the differences lie within this range. Good reliability was observed between ODI4_RING_ and both AHI4_PSG_ and ODI4_PSG_ measurements (ICC = 0.84–0.85). Correlations between ODI4_RING_ and both AHI4_PSG_ and ODI4_PSG_ were strong (*r* = 0.85–0.87), with an RMSE of 6.6 and 7.1 events/h, respectively, meaning that the standard deviation of the difference between the observed and predicted values was less than the difference between mild and moderate OSA classification (10 events/h). The ROC curves showed that the Ring can identify moderate-to-severe sleep apnea per AHI4_PSG_ criteria with excellent discriminative value (AUC = 0.84), with an optimal threshold cutoff selected of ODI4_RING_ = 10.7 events/h for high sensitivity (0.98) but lower specificity (0.60). The optimization model was preferentially weighted for high sensitivity to maximize the number of true positives included, considering the intended application of the Ring as a prescreening tool; however, in different situations, alternative optimal operating points on the ROC curve could be chosen to increase specificity at the cost of lower sensitivity, for example, to make treatment decisions.

We think that the observed underestimate in ODI4 with the Ring vs. PSG is predominately attributable to the use of the total recording time as a denominator in the Ring ODI4 calculations vs. total sleep time for PSG metrics: Indeed, in exploratory analysis, using PSG total sleep time instead as the denominator for Ring ODI4, we instead see a small positive mean bias (+2.8 events/h with ODI4Ring vs. ODI4PSG; see Supplementary Figure S2), suggesting that the Ring could, in fact, be more sensitive to oxygen desaturations than the PSG finger probe if sleep time is better accounted for. A greater bias at higher ODI4 values is not seen in a sleep-time-adjusted analysis. Sensor placement at the fingertip with the PSG probe vs. the base of the finger using the Ring may also contribute to differences. Nevertheless, systematically underestimating OSA severity may have important clinical implications on diagnosis or classification if not accounted for in the interpretation of metrics, for example, by using adjusted thresholds. We note that the optimal screening cutoff for moderate to severe OSA identified using ODI4_RING_ is lower than the standard AHI criteria (10.8 vs. 15) and still produced excellent discrimination.

We also have demonstrated for the first time that it is feasible to calculate HB from a stand-alone wearable device using the oximetry signal alone. HB_RING_ and HB_PSG_ showed moderate-to-good agreement (ICC = 0.73). Hypoxic burden has been shown to be sensitively associated with a greater risk of cardiovascular disease and mortality (Azarbarzin et al., 2018, 2020), adding to the increasing evidence that intermittent hypoxemia plays a key role in the systemic long-term physiological consequences of OSA. Thus, in the future, monitoring oximetry-based metrics may be very useful for predicting disease risk and stratifying those who may most benefit from treatment.

The findings presented here are comparable with other wearable oximetry devices. A recent systematic review showed considerable variability in oximeters' performance across studies, with mean differences between oximetry to AHI_PSG_ that ranged from −13.7 to 4.8 events/h (Khor et al., 2023) and sensitivity and specificity of ODI values that ranged from 49 to 97% and 64 to 100%, respectively, for classifying AHI_PSG_ >15 events/h (Khor et al., 2023). A wireless finger-worn oximeter and cloud-based analysis system (Oxistart, Biologix Sistemas Ltd., Brazil) were shown to accurately detect OSA, with a mean difference of 2.9 events/h and limits of agreement of ±16.5 events/h compared to AHI4_PSG_ and an AUC 0.96 classifying moderate-to-severe OSA (Pinheiro et al., 2020). However, direct comparisons are challenging given differences in populations, OSA diagnostic criteria, ODI thresholds, and the selected optimal operating threshold between studies. The WatchPAT (Itamar Medical Inc., Caesarea, Israel) measures peripheral arterial tonometry in addition to oximetry, heart rate, and actigraphy and was found to detect AHI > 15, with an average sensitivity and specificity of 92.21 and 72.39%, respectively, in a recent meta-analysis of previous evaluation studies, with considerable variability between studies (Iftikhar et al., 2022). A commercial wrist-worn oximetry device (Galaxy Watch 4, Samsung, South Korea) can distinguish moderate-to-severe OSA (AUC: 0.80–0.91); however, device data rejection rates were high (26.5–52.3%; Jung et al., 2022; Browne et al., 2024).

Oximetry monitoring using a wearable device also provides specific advantages over the current gold standard, PSG. Unlike PSG, with many channels to precisely monitor multiple parameters across one night, the Ring could easily be used over multiple nights at home to provide accurate, multi-night, and averaged metrics to follow changes over time, for example, to assess treatment efficacy. The substantially lower cost of an oximetry device compared to a full PSG system allows for its use in a broader range of settings, including underserved communities and lower-income countries, where a full PSG is not feasible. Currently, night-to-night variability is recognized as a significant source of inconsistent measurement of OSA within a patient and can often lead to misdiagnosis (Roeder et al., 2020; Punjabi et al., 2020). Repeated measurements of OSA severity to reduce the effects of night-to-night variability correlate better with adverse cardiovascular outcomes than with single-night measures and reduce the misdiagnosis of OSA (Lechat et al., 2023). Algorithmically identifying events also avoids uncertainty introduced by the inter-scorer variability in human scoring of AHI measures (Thomas et al., 2020; Collop, 2002). These sources of variability are also inherent in current single-night HST approaches to quantifying residual OSA severity on therapy and could potentially be mitigated through unobtrusive multi-night oximetry to get a more accurate representation of the true ongoing therapeutic response. Providing clinicians and/or patients with simple objective measurements of treatment efficacy or disease progression over time could help inform decisions about the best management strategies on an individualized basis.

It is known that oximetry-based metrics, including ODI and hypoxic burden, may be systematically underestimated in people with darker skin due to an underestimation of oxygen desaturation using pulse oximetry (Sjoding et al., 2020). The current study was not sufficiently powered to perform a formal analysis of the effects of skin pigmentation on the reliability of oximetry metrics. However, we note that the average bias was similar across the groups in our study (Table 2). Future studies are required to better explore this in a large diverse cohort.

**Table 2:** Measurement bias by Fitzpatrick scale subgroup.

**Fitzpatrick scale**	**AHI4_PSG_ – ODI4_RING_ (events/h)**	**ODI4_PSG_ – ODI4_RING_ (events/h)**	**HB_PSG_ – HB_RING_ (%min/h)**
I, II (*N* = 15)	0.2 [−3, 7.6]	1.7 [−1.6, 7.5]	1.8 [−12.4, 17]
III, IV (*N* = 6)	1.7 [−1.8, 5.5]	2.8 [0.0, 5.5]	3 [−8, 27]
V, VI (*N* = 4)	1.6 [−11.1, 4.6]	2.3 [−2.2, 5.5]	−1.8 [−20.5, 20]

Data are presented as median [interquartile range]. The Fitzpatrick scale is a numerical classification schema for human skin color, where I is the palest and VI is the darkest tone.

AHI4_PSG_, apnea–hypopnea index from PSG (hypopneas scored by >4% desaturation criteria); ODI4_PSG_, oxygen desaturation index from PSG (hypopneas scored by >4% desaturation criteria); ODI4_RING_, oxygen desaturation index from Ring oximeter (hypopneas scored by >4% desaturation criteria); HB_PSG_, hypoxic burden from PSG; HB_RING_, hypoxic burden from ring oximeter.

Including other clinical features or spectral or non-linear characteristics of the oximetry signal into a multivariate classifier of OSA could further improve performance as a screening tool (Terrill, 2020; Behar et al., 2019), but the current study sought to limit the complexity of this initial validation study. In general, multivariate models have shown marginal improvements in accuracy compared to classifying with ODI alone (Uddin et al., 2018).

### 4.1 Strengths and limitations

This study was performed in a sleep laboratory under controlled conditions; metrics determined by the Ring device at home in an uncontrolled environment may exhibit greater variability compared with more controlled conditions. However, it could be argued that measurements made using minimally disruptive equipment in a usual sleep environment may be a better representation of true disease severity. Oximetry-based monitoring uses the total recording time as the denominator to assess metrics compared to total sleep time used in a PSG study with simultaneous EEG recording, which likely contributes to the average underestimate of oximetry metrics from the Ring. Measurement bias may be greater in those with reduced sleep efficiency, for example, patients with comorbid insomnia or other complex sleep disorders. Ongoing work to determine sleep–wake staging using Ring accelerometry, oximetry, and pulse data could help address these concerns. Our study is a proof-of-concept trial conducted as part of a separate clinical trial; therefore, we did not perform *a priori* sample size calculations. *Post-hoc* power simulations indicated the current sample size provided a power of 0.991 to detect a clinically meaningful effect of 5 events/h (based on the median absolute difference between two repeated gold-standard PSG measurements of OSA severity; see the Supplementary material for details). Nevertheless, further studies with larger sample sizes across diverse populations are warranted to validate these findings. This study involved a prescreened population of OSA patients under different therapeutic conditions, only 10/90 PSGs exhibited no OSA per the gold-standard assessment (AHI4 < 5). We think that this is representative of a target population for the intended use of continued monitoring of OSA and in individual responses to therapy; however, it is likely not reflective of a screening population. Including more non-OSA control subjects, that is, better reflecting a community cohort, would better describe the performance for a target use for screening, and may result in different, potentially improved, sensitivity and specificity of the device. The ROC optimal operating thresholds may be different in this population, where more subjects are expected to have low AHI. Finally, the oximetry metrics explored in this study do not distinguish between central and obstructive respiratory events, whose gold-standard classification requires measuring respiratory effort. The diagnostic accuracy from the Ring alone may be lower in those with complex or central sleep apnea. The current findings may also not be representative of OSA patients with excluded comorbidities (e.g., significant cardiac disease or uncontrolled hypertension). However, pulse oximetry could still be used as a useful screening tool that could help clinicians identify those that may require a full PSG, and future algorithm development could enhance the ability to predict central events from obstructive events using event-based features.

### 4.2 Conclusion

Ring oximetry measures of OSA severity showed strong correlations with current gold-standard PSG criteria and were able to identify moderate to severe OSA with excellent discriminative value. This study shows the Ring oximeter has substantial promise as an accessible tool for the identification and multi-night monitoring of OSA severity.

## Data Availability

The datasets presented in this article are not readily available because data sharing requests will be considered from research groups that submit a research proposal and an appropriate statistical analysis plan and dissemination plan. Data will be shared via a secure data access system. Requests to access the datasets should be directed to ltaranto@apnimed.com.
