# Supplementary material for: Performance evaluation of a ring-worn pulse oximeter for the identification and monitoring of obstructive sleep apnea
Source: Front Sleep. 2025 Apr 15;4:1549272. doi: 10.3389/frsle.2025.1549272 (PMC12713970; doi:10.3389/frsle.2025.1549272)
Supplement: Supplementary file 1 [file Data_Sheet_1.docx]

**Performance evaluation of a ring-worn pulse oximeter for the identification and monitoring of obstructive sleep apnea**

Laura K. Gell^1^, Ketan Mehta^1^, Neda Esmaeili^2^, Luigi Taranto-Montemurro^1^, Scott A. Sands^2^, Stephen D. Pittman^1^, Ali Azarbarzin^2^.

^1^ Apnimed Inc., Cambridge, MA

^2^Division of Sleep and Circadian Disorders, Brigham and Women's Hospital and Harvard Medical School, Boston, MA

**Supplementary Discussion**

We conducted a simulation-based power analysis to assess the adequacy of the sample size for detecting the primary outcome of our generalized mixed model analysis, the bias between the Ring and PSG ODI4 measurements. We defined a clinically meaningful effect size using the median absolute night-to-night variability between two repeated baseline PSG measurements using data from a previous phase 2 clinical trial (5 events/hr, 292 patients, (1)). Using the observed variances and residuals from our primary model, we simulated datasets with the current sample size of 25 patients and 90 observations, and refitted the analysis model for each simulated dataset. Across 1000 iterations, we assessed the significance of the main method difference term (Ring vs PSG) and determined the proportion of iterations that were significant. The results demonstrated a high statistical power of 0.991 to detect a 5 events/hr difference between the two methods. Furthermore, we simulated the effect of varying sample sizes and included a power curve figure (Supplemental Figure 2), which illustrates that increasing the sample size would not substantially improve power for detecting the effect. This analysis supports the adequacy of the chosen sample size for the study’s objectives, though larger studies across more diverse populations would be valuable to confirm these findings.

**Supplementary Figures**

Supplementary Figure 1. Correlation (left) and Bland-Altmann (right) plots to compare adjusted ODI4_RING_ and ODI4_PSG_, where has been calculated as events per hour of total sleep time, using the scored sleep time from PSG. Note that bias is now positive, i.e. the ODI4 values from the Ring are now slightly higher than from PSG on average, and there is no increase in bias with higher ODI4 values.


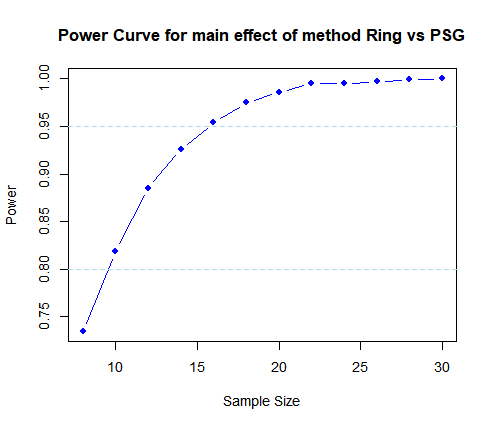
Supplementary Figure 2. Power curve to detect a main effect of method (Ring vs PSG) on ODI4 at a clinically significant threshold of 5 events/hr, using mixed model analysis of simulated data sets. Sample size refers to number of simulated patients, assuming each provides 4 observations under 4 different treatment conditions (for example, a sample size of 20 results in 80 simulated observations).

**Supplementary References**

1. Schweitzer, P.K., Taranto-Montemurro, L., Ojile, J.M., Thein, S.G., Drake, C.L., Rosenberg, R., Corser, B., Abaluck, B., Sangal, R.B. and Maynard, J., 2023. The combination of aroxybutynin and atomoxetine in the treatment of obstructive sleep apnea (MARIPOSA): a randomized controlled trial. *American journal of respiratory and critical care medicine*, *208*(12), pp.1316-1327.
